# Serological Cross-Reactivity Between *Bovine alphaherpesvirus 2* and *Bovine alphaherpesvirus 1* in a gB-ELISA: A Case Report in Italy

**DOI:** 10.3389/fvets.2020.587885

**Published:** 2020-10-28

**Authors:** Stefano Petrini, Patricia König, Cecilia Righi, Carmen Iscaro, Ilaria Pierini, Cristina Casciari, Claudia Pellegrini, Paola Gobbi, Monica Giammarioli, Gian Mario De Mia

**Affiliations:** ^1^National Reference Laboratory for Infectious Bovine Rhinotracheitis (IBR), Istituto Zooprofilattico Sperimentale Umbria-Marche “Togo Rosati”, Perugia, Italy; ^2^OIE and National Reference Laboratory for Bovine Herpesvirus Type 1 Infection, Friedrich-Loeffler-Institut, Greifswald, Germany

**Keywords:** Calf, BoHV-2, BoHV-1, serological cross-reactivity, performance test station

## Abstract

In this study, we demonstrated for the first time in Italy, the serological cross-reactivity between *Bovine alphaherpesvirus 2* (BoHV-2) and *Bovine alphaherpesvirus 1* (BoHV-1). Five months after arriving at a performance test station in Central Italy, a 6-month-old calf, which was part of a group of 57 animals, tested positive for BoHV-1 in a commercial gB-ELISA test. It was immediately transferred to the quarantine unit and subjected to clinical observation and serological and virological investigations. During this period, the calf showed no clinical signs. The results from laboratory investigations demonstrated the presence of antibodies via competitive glycoprotein B (gB) ELISAs, indirect BoHV-1 ELISAs, and indirect BoHV-2 ELISAs. Furthermore, the plaque reduction assay provided evidence for the presence of antibodies only for BoHV-2, whereas the virus neutralization test showed negative results for both BoHV-1 and BoHV-5. These findings strongly suggest the occurrence of a serological cross-reactivity between BoHV-2 and BoHV-1. Interference of BoHV-2 antibodies in serological BoHV-1 diagnostics should be considered during routine IBR tests, especially when animals are kept in a performance test station.

## Introduction

*Bovine alphaherpesvirus 2* (BoHV-2) is a member of the family *Herpesviridae* and belongs to the genus *Simplexvirus* (1). The virus was first isolated from a cattle with skin infection on a farm called Allerton in 1957 in South Africa. The aetiological agent is associated with two different clinical forms, a localized skin disease named bovine mammillitis, bovine herpes mammillitis, or bovine ulcerative mammilitis and a generalized disease called Pseudo-Lumpy Skin Disease (PLSD). BoHV-2 infection has been reported in Africa (South Africa, Kenya, Tanzania, Rwanda-Burundi), Europe, the United States, and Australia (2–5). Recently, the virus was isolated from a clinical case of PLSD in northern Italy (6). However, there are very limited data available on the serological evidence of the virus in Italian cattle farms (7). A serological cross-reactivity has been observed between BoHV-2 and *Bovine alphaherpesvirus 1* (BoHV-1), (5, 8). This phenomenon could lead to severe consequences in BoHV-1 serology, resulting in incorrect diagnosis of BoHV-1, both in areas where there are active control/eradication plans for Infectious Bovine Rhinotracheitis (IBR) and in performance test stations. Moreover, BoHV-2 is similar to BoHV-1 in that it can establish viral latency and be reactivated following an immunosuppressive stimulus, leading to the spread of the virus throughout the herd, causing potential economic losses (9).

In this study, we report, for the first time, the occurrence of serological cross-reactivity between BoHV-2 and BoHV-1 in a calf detained at a performance test station located in Central Italy.

## Case Description

A 6-month-old beef calf (Id. 365/29-04), asymptomatic and seronegative for BoHV-1, was introduced into a performance test station located in Central Italy in October 2018. Following the due protocol for the evaluation of morphological and genetic characteristics, the animal was initially quarantined for 30 days. Two consecutive serum samples were taken 24 days apart. The samples were tested for antibodies against glycoprotein B (gB) of BoHV-1 using a commercial competitive ELISA test (gB-ELISA). They were also tested for neutralizing antibodies against BoHV-1 using virus neutralization (VN) test. The protocol of performance test station does not include investigations against Bovine alphaherpesvirus 2 (BoHV-2). Further, upon testing negative for both the antibodies (gB, VN), the animal was introduced into a group of 56 calves of the same age. These animals were selected from different cattle farms know to be IBR free. Serum and blood samples were taken from all the animals, on a monthly basis, for serological and virological investigations of BoHV-1. The serum samples were tested for the specific antibody via competitive gB-ELISA and VN test. In addition, the EDTA blood samples were used for the detection of BoHV-1 DNA via real-time PCR.

The competitive gB-ELISA test was carried out using the protocol provided by the kit, and the results were expressed according to manufacturer's instructions. VN test and real-time PCR were performed according to the protocols described in the OIE Manual of Diagnostic Tests and Vaccines for Terrestrial Animals (10). All the animals tested negative until February 2019.

In March 2019, the above-mentioned calf (Id. 365/29-04) tested positive in the competitive gB-ELISA test. Although, no clinical IBR symptoms were observed, the animal was immediately placed in quarantine for 30 days. Clinical observations were performed on a daily basis and further serological and virological investigations were carried out. In particular, nasal swabs and EDTA blood samples were collected for virus isolation and real-time PCR, respectively, following the procedures described in the OIE Manual of Diagnostic Tests and Vaccines for Terrestrial Animals (10).

The serum samples were tested for BoHV-1 using different commercial ELISAs: (i) competitive gE-ELISA (A, B, C); (ii) competitive gB-ELISA (D, E), and (iii) indirect-ELISA (F, G, H, I). Additionally, we also performed plaque reduction assay and VN test against BoHV-1.

In order to assess any serological cross-reactivity with other herpesviruses, the serum samples were tested for antibodies against the following aetiological agents: (i) *Bovine alphaherpesvirus 2* (BoHV-2), (ii) *Bovine gammaherpesvirus 4* (BoHV-4), (iii) *Bubaline alphaherpesvirus 1* (BuHV-1), and (iv) *Bovine alphaherpesvirus 5* (BoHV-5). Different indirect ELISA tests were employed to detect BoHV-2 (L), BoHV-4 (M), and BuHV-1 (N). Further, plaque reduction assay and VN test was performed against BoHV-2 and BoHV-5, respectively. The presence of BoHV-2 genome was surveyed via PCR using blood samples.

The ELISA tests were performed following the protocols provided by the kits and the results were expressed according to manufacturer's instructions. Additionally, for the plaque reduction assay BoHV-1 strain Schönböken and BoHV-2 strain RVB 0064 (Biobank, Friedrich-Loeffler-Institut, Insel Riems, Germany) were adjusted to 25–50 plaque forming units (pfu) per 50 μl. Sera were subjected to one freeze-thaw cycle followed by heat inactivation for 30 min at 56°C. Further, 50 μl of 2-fold serially diluted serum was incubated with the test virus for 24 h at 37°C to enable virus neutralization. The serum-virus suspensions were inoculated onto 1-day old Madin-Darby Bovine Kidney cells (1.25 × 10^5^ cells per well). The cells were obtained from the collection of cell lines in veterinary medicine (CCLV, FLI, Insel Riems, Germany), identified by the code MDBK-261. After incubating for 1 h at 37°C, supernatants were removed and replaced with semi-solid overlay medium containing 0.25% methylcellulose (11). Plaque counts were determined 3 days later. Titres were defined as highest dilutions that induced relevant neutralization (≤50% of control values).

The VN test for BoHV-5 was performed on 96-well-tissue culture microtiter plates using the NA67 strain of the virus. Sera were heat-inactivated at 56°C for 30 min. Briefly, 50 μl of each 2-fold serial dilutions were mixed with 50 μl of 100 TCID_50_ of virus in duplicates. The plates were incubated at 37°C and 5% CO_2_ for 1 h, and then MDBK cells were seeded at a density of 30,000 cells/well (100 μl). The cells were provided by Biobanking of Veterinary Resources (BVR, Brescia, Italy) and identified by the code BS CL 63. Readings were taken after 72 h, when the cytopathic effect was complete in virus positive control cultures. The titer of each serum was expressed as the highest dilution neutralizing the virus. The BoHV-2 genome was detected using a protocol described by De Giuli et al. (12).

## Results

No clinical signs were observed in the calves during the quarantine period. The serological results are shown in Table 1. The calf (Id. 365/29-04), tested seropositive in 1 out of 2 competitive gB-ELISAs and in 2 out of 4 indirect-ELISAs. BoHV-2 antibodies were also detected via indirect ELISA. However, no seropositivity was observed in competitive gE-ELISA and indirect BoHV-4 and BuHV-1 ELISAs. Additionally, the plaque reduction assay provided evidence for a positive result only for BoHV-2, with a mean antibody titer of 1:384, while the VN assay showed no evidence for BoHV-1 and BoHV-5. The virological investigations were consistently negative.

**Table 1:** Antibody response obtained from different ELISA tests against BoHV-1, BoHV-2, BoHV-4, and BuHV-1 in the serum sample obtained from a performance station in Central Italy.

**ELISA**											
**BoHV-1**									**BoHV-2**	**BoHV-4**	**BuHV-1**
Competitive gE-ELISA			Competitive gB-ELISA		Indirect-ELISA				Indirect-ELISA	Indirect-ELISA	Indirect-ELISA
A	B	C	D	E	F	G	H	I	L	M	N
–	–	–	+	–	+	+	–	–	+	–	–

## Discussion

In this study, we reported a case of serological cross-reactivity between BoHV-2 and BoHV-1 in a calf detained in a performance test station in Central Italy. BoHV-2 infections have also been described in Africa, Europe, the United States, and Australia (2–4). Several European countries have reported unexplained cases of gB-positive singleton reactors and they were found to be gE-negative (5, 8, 13, 14).

In this report, we have shown that 1 out of 2 commercial competitive gB-ELISAs gave a positive result which was not confirmed by BoHV-1 plaque reduction assay, VN, or competitive gE-ELISA tests. These serological results were inconsistent with immune responses usually developed by a BoHV-1 infected animal (15–17). Antibodies against glycoprotein B of BoHV-1 or neutralizing antibodies appear after 7–14 days post-infection, increase at constant levels, and persist for long periods. In contrast, antibodies against glycoprotein E (gE) appear 30–35 days post-infection and also persist for long periods (18, 19). However, Mars et al., reported that non BoHV-1 related gB-singleton reactors were found to be negative in the gE-ELISA test. Our study showed that, the calf detained at the performance station tested negative for all the three gE-ELISA tests. This was in concordance with the findings of previous studies (5, 8, 13). Increase in gE-reactivity was not detected over a period of 3 months. Seroconversion for gE would be expected in unvaccinated animals within this timespan.

The results obtained in this study could be attributed to non-specific reactivity, as indicated by Beer et al., such as batch variation between ELISA kits, sample quality, or the use of fresh serum (20). However, all of these factors have been taken into consideration in this study. Furthermore, different studies have shown that the seropositivity of some animals in competitive gB-ELISA could be attributed to serological cross-reactivity with other ruminant alphaherpesviruses (5, 8). This antigenic relationship has been demonstrated using different diagnostic tests (5, 21). In particular, the epitopes responsible for the cross-neutralization are located in the major glycoprotein gB, gC, and gD (22). The gB gene is the most conserved among the major herpesvirus glycoproteins (23, 24). In this context, we investigated potential cross-reactivity of BoHV-1 with the following viruses: *Bovine alphaherpesvirus 2* (BoHV-2), *Bovine gammaherpesvirus 4* (BoHV-4), *Bovine alphaherpesvirus 5* (BoHV-5), and *Bubaline alphaherpesvirus 1* (BuHV-1).

Our results demonstrated that indirect-ELISA detected antibodies against BoHV-2 and this was subsequently confirmed via plaque reduction assay and BoHV-2 neutralization assay. The sanitary protocol of the experimental station, does not efficiently control BoHV-2 infection. Thus, the calf was not serologically checked for this viral infection while entering the experimental station.

It is well-known that reactivation is typical of herpesviruses and generally occurs after an immunosuppressive stimulus (25) or after dexamethasone treatment (9). We speculated that the serological cross-reactivity detected 5 months after arriving resulted from the latency state in a calf passing the first infection, rather than a subclinical primary infection. This hypothesis is also supported by the fact that if a primary infection had occurred after the entrance of the calf into the performance station, other animals had to show clinical signs, and then seroconverted against BoHV-2 as well, consequently some more animals might have been identified by gB-ELISA IBR tests. In addition, BoHV-2 spread might not be efficient in this herd (insect control, no role of milking cluster). Additionally, the performance station benefits from a very high biosecurity level, as it is located in an isolated area and is accessed only by personnel dedicated to the activities of the station. Therefore, an accidental entry of wild-type virus is most unlikely.

Furthermore, as the performance station is equipped with traps for biting flies, BoHV-2 transmission by flies may be excluded. This leads us to conclude that a latent BoHV-2 virus might have been reactivated in the calf as a consequence of an immunosuppressive stimulus, possibly when the animal underwent a change of diet or after its introduction into the performance station group. Unfortunately, as required by performance station regulations, the other animals, all asymptomatic and seronegative to BoHV-1 tests, were separated and sold during the study period. Thus, it was not possible to conduct further investigations on the cohabiting calves. However, the seropositivity ascertained in the calf cannot be attributed to vaccination because (i) the health regulations to regarding access a performance station ban the introduction of animals vaccinated against IBR and (ii) there are no commercially available vaccines against BoHV-2. Moreover, according to their regulations, animals entering a performance station are selected from IBR free herds, for which the practice of vaccination is prohibited. The detection of singleton reactors is crucial for the selection of animals in a performance test station, where animals can be introduced only if antibody negative. Additionally, each animal is checked every month to verify that no latent viral infections are reactivated.

Furthermore, in the context of IBR eradication programs, it is important to accurately identify singleton reactors. As an example, in Italy, where an active plan for the eradication of BoHV-1 in beef cattle breeds is in place (26), 20 gB singleton reactors were evidenced in different regions, during the 2018–2019 campaign (data not shown).

## Conclusions

In conclusion, the present study highlights latent reactivation of BoHV-2 in a calf, which confirmed serological cross-reactivity with different commercial BoHV-1 ELISA tests. This should be carefully taken into consideration, when uncertain interpretation of IBR serology occurs, especially in performance test stations, where accidental contact to vaccine virus or wild type BoHV-1 infection can be reliably ruled out. In fact, animals erroneously considered as positive for BoHV-1, could be eliminated needlessly, which concomitantly means losing an animal of high genetic and economic value. In addition, the cross-serological reactivity may have an economic and social impact on control and eradication programs (trade restrictions, loss of negative status, decline in acceptance).

## Data Availability Statement

All datasets presented in this study are included in the article/supplementary material.

## Ethics Statement

In this study, all data analyzed were collected as a part of the routine diagnosis, therefore, according to the national legislation, ethics approval, and written informed consent are not required.

## Author Contributions

Experimental conception and design were done by SP. Collection of samples was done by CR. Immunological analyses were done by CP, CC, PG, and IP. Analysis, interpretation was done by SP, PK, CR, CI, and MG. Paper writing and editing were done by SP, PK, and GD. All authors read and approved the final manuscript.

## Conflict of Interest

The authors declare that the research was conducted in the absence of any commercial or financial relationships that could be construed as a potential conflict of interest.
